# Refugee mental health research: challenges and policy implications

**DOI:** 10.1192/bjo.2020.90

**Published:** 2020-09-03

**Authors:** Lucia Chaplin, Lauren Ng, Cornelius Katona

**Affiliations:** South London and Maudsley NHS Foundation Trust, UK; University of Essex, Colchester, UK; Helen Bamber Foundation, London; and Division of Psychiatry, University College London, UK

**Keywords:** Refugee, asylum seeker, mental health, epidemiology, research implications

## Abstract

Mental illness is common among forced migrant populations, and ongoing mental illness can hinder refugees’ ability to negotiate the asylum process. This editorial rehearses the challenges of undertaking research among forced migrant populations, exploring how they could be addressed in future research, and outlines differences between forced migrant groups. It points to the growing body of evidence that can be called on in advocating for systemic change in government policy and mental health services, with significant support for a sensitive and objective inquisitorial approach to gathering evidence in support of asylum claims.

The world currently hosts the highest number of refugees since the Second World War. With nearly 80 million forcibly displaced, 26 million of whom hold refugee status,^1^ the needs of refugees and asylum seekers have become an increasing concern for mental health services. Although the experience of each refugee and asylum seeker will differ, traumatic events and ongoing stress are often prominent. As a result, research into the mental health of refugees and asylum seekers has expanded.

Henkelmann et al^2^ have made an important contribution to this research by providing an up-to-date overview of the prevalence of mental illness among forced migrants. Their intention was, in part, to highlight some of the heterogeneities among studies conducted in this arena, in addition to addressing the challenges faced in researching this population. Their work indicates that mental illness is common among forced migrant populations and adds to the body of evidence that can be called on in advocating for systemic change in government policy and mental health services.

## Challenges of researching forced migrant populations

There are many complexities in carrying out cross-culturally informed research with forced migrants. Personal narratives, vulnerabilities, barriers to access and cultural perspectives on mental illness have been recognised as some of the difficulties in this field of research, particularly among refugees and asylum seekers.

### Disclosure of personal information

There is a pervasive ‘culture of disbelief’ in Western asylum systems, whereby those claiming asylum are required to ‘prove’ their eligibility for legal protection. In the absence of personal documents (which are frequently left behind or lost during flight), asylum claims commonly rely on personal accounts. These claims are often heavily interrogated and scrutinised for any inconsistencies.^3^ As a result, the sharing of a story becomes a means through which safety is guaranteed or rejected. It is difficult for immigration officials, lawyers and clinicians carrying out their assessments to ensure that consent is fully informed and free from coercion.^4^ We have recently reported on the ‘narrative dilemmas’ asylum seekers often face in the process of recounting the experiences that led them to leave their countries of origin as well as during their asylum journeys.^5^

During the process of seeking refugee status, the expression of vulnerability can take on different meanings. Asylum seekers may need medical certification, providing detailed accounts of their physical and psychological symptoms, to ‘verify’ their claim of torture. However, ongoing mental illness may hinder their ability to recount their trauma because such disclosures can be re-traumatising. This may have an adverse impact on their asylum claim as well as leading to underreporting of mental health problems in research studies.

### Cultural interpretations of mental illness

It has also been argued that Western classifications of mental illness have an inappropriately ethnocentric focus. As responses to trauma vary across cultural and ethnic backgrounds, it can be difficult to interpret manifestations of mental illness universally owing to the lack of a nomenclature for trauma that is valid across cultures.^6^ Thus, tools used for identifying groups who need specialist mental health attention may not be adequate among those from different cultural groups. This can make it difficult for clinicians to identify and diagnose those who may need specialist treatment and to make acceptable and effective care plans.^7^

## The implications of migration status

Henkelmann et al^2^ were unable to break down the available data by migration status, and instead used a broad and non-legal definition of ‘refugee’. It may be argued that anyone who flees a country needs refuge and is therefore a ‘refugee’. Assigning a legal label is therefore a political process and may be of little relevance in the context of mental illness.

However, we would argue that data relating to migration status are vital in future research in this area, as they allow us to investigate the specific mental health impacts of asylum policies. In this section, we will expand on some of the crucial differences between forced migrant groups.

### Threat of return

In international law, a refugee is defined as a person who:

‘owing to well-founded fear of being persecuted for reasons of race, religion, nationality, membership of a particular social group or political opinion, is outside the country of his nationality and is unable or, owing to such fear, is unwilling to avail himself of the protection of that country’.^8^

This remains a narrow legal definition, which excludes categories of people who have been displaced outside of these boundaries, for instance those fleeing natural disasters. Nevertheless, it confers a certain set of rights on the individual, including the right to live and work in the host country and subsequently to apply for permanent residence (in the UK, indefinite leave to remain (ILR)) or citizenship. For those fleeing generalised conflict or indiscriminate violence, a similar status of ‘humanitarian protection’ may be given. These statuses are both time-limited, and it is only when individuals are granted permanent residence/ILR or citizenship that their uncertainty over whether they will be forced to return to their country of origin can end. This may contribute to the findings of Henkelmann et al^2^ that length of stay appeared to be unrelated to prevalence of mental illness. As the authors note, the findings may be different if broken down by whether permanent status was granted.

An asylum seeker is anyone who has arrived in a host country and made an asylum claim. In the UK, while their case is being reviewed, they have the right to remain and are provided with a basic level of housing and subsistence, but (with very few exceptions) are not allowed to work. This existence ‘in limbo’, with the constant threat of imminent return, can represent a significant trauma in addition to their experiences in their country of origin and may increase their vulnerability to mental illness and impede recovery.^9^

### Traumatic journeys and post-migration factors

Less than 1% of refugees arrive in their host country through a resettlement programme in which they are given refugee status prior to arrival.^10^ These more favourable circumstances allow refugees to benefit from a greater degree of stability and certainty regarding their immigration status, along with the right to work or to claim mainstream benefits immediately.^11^

However, owing to the highly restricted availability of this route, many asylum seekers and refugees make long and risky journeys, and may arrive in their host country after having been exposed to additional trauma, such as trafficking or destitution, during their journey. This may leave them more vulnerable to a traumatic asylum process.

Henkelmann et al's study rightly makes reference to some of the post-migration factors that can affect mental health, including migration status, socioeconomic position, long-drawn asylum procedures, unemployment and discrimination. These factors may also contribute to a range of mental health problems, including significantly higher rates of psychosis,^12^ a diagnostic group that was not included in the authors’ analysis.

Another diagnosis whose prevalence the study did not examine is ‘complex post-traumatic stress disorder’ (complex PTSD). This diagnostic concept, which is characterised by ‘disorders of self-organisation’ (impaired relationships, emotional dysregulation and negative self-concept) as well as core PTSD symptoms, has now been incorporated into ICD-11.^13^ Complex PTSD is often found in the aftermath of multiple and repeated trauma^14^ – such as the torture, modern slavery, human trafficking and sexual abuse experienced by many asylum seekers.

The restrictive asylum systems in operation in most Western countries mean that, in addition to threat of return, asylum seekers have no right to work, and live in poor housing with minimal monetary funds for subsistence.^11^ Many are placed in immigration detention, a process likely to have an adverse impact on their mental health.^15^ Furthermore, despite social networks being recognised as key sources of support for asylum seekers, dispersal policies in the UK have made it increasingly difficult for asylum seekers and refugees to integrate into a community, in addition to making clinical and research follow-up more challenging.

Many forced migrants remain undocumented and therefore largely invisible to healthcare and other support services. Despite legal entitlement to primary healthcare services, many asylum seekers struggle to register with general practitioners owing to a lack of a fixed address, leading to substandard management of long-term health conditions. The COVID-19 pandemic has also served to highlight the precariousness of their situation, given the current need for testing and tracing as part of a public health response.

Within the Organisation for Economic Cooperation and Development (OECD), Canada has an explicitly inquisitorial asylum system.^16^ In Europe, a lack of common standards in asylum policies has led to varied treatment between countries, many of which are adversarial and unwelcoming in their approach to refugees and asylum seekers. However, one example to be commended is Germany's response to the large influx of Syrian refugees to Europe in 2015, wherein the government decided to accept a comparatively high number of asylum seekers as part of an organised resettlement programme for Syrians fleeing conflict. According to statistics published by the World Bank, in 2019 Germany remained the European country with the highest number of refugees (1.1 million, compared with the next highest country, France, with 400 000), with the UK hosting roughly 130 000 refugees.^17^

## Conclusions

Henkelmann et al have collated clear and compelling evidence of the high rate of mental illness among forced migrants. Their research has also highlighted that undertaking research among forced migrant populations can be challenging. Such research deserves prioritisation.

Mental illness itself may be a factor impeding recognition of the need for and entitlement to protection. In countries where an adversarial asylum policy is in place, in which asylum seekers are required to prove their entitlement against the host country's attempt to disprove it, the presence of a mental health condition will likely amplify the unfairness of an already hostile system and risk their ability to gain refugee status.

Henkelmann et al's study therefore provides significant support for the contention that, as we have previously argued,^9^ a fair asylum policy for vulnerable people, utilising an inquisitorial approach where evidence can be gathered in a sensitive and objective manner, is crucial for a fair asylum process.

## Author contributions

All authors were involved in developing the concept and planning. L.C. drafted the majority of the manuscript, with some sections drafted by L.N. L.N. and C.K. completed edits, and all authors reviewed and approved the final version of the manuscript.

## Declaration of interest

C.K. reports receiving a salary from the Helen Bamber Foundation outside the submitted work.

### Supplementary material

For supplementary material accompanying this paper visit http://dx.doi.org/10.1192/bjo.2020.90. click here to view supplementary material
